# Correlation between thrombocytopenia and host response in severe fever with thrombocytopenia syndrome

**DOI:** 10.1371/journal.pntd.0008801

**Published:** 2020-10-29

**Authors:** Xiao-Kun Li, Ke Dai, Zhen-Dong Yang, Chun Yuan, Ning Cui, Shao-Fei Zhang, Yuan-Yuan Hu, Zhi-Bo Wang, Dong Miao, Pan-He Zhang, Hao Li, Xiao-Ai Zhang, Yan-Qin Huang, Wei-Wei Chen, Jiu-Song Zhang, Qing-Bin Lu, Wei Liu

**Affiliations:** 1 State Key Laboratory of Pathogen and Biosecurity, Beijing Institute of Microbiology and Epidemiology, Beijing, P. R. China; 2 The 990 Hospital of Chinese People's Liberation Army Joint Logistic Support Force, Shihe District, Xinyang, P. R. China; 3 The Shangcheng Center for Disease Control and Prevention, Shangcheng County, Xinyang, P. R. China; 4 Treatment and Research Center for Infectious Diseases, the Fifth Medical Center of Chinese PLA General Hospital, Fengtai District, Beijing, P. R. China; 5 Department of Laboratorial Science and Technology, School of Public Health, Peking University, Haidian District, Beijing, P. R. China; 6 Key Laboratory of Vector Borne and Natural Focus Infectious Diseases, Beijing, People’s Republic of China; Saudi Ministry of Health, SAUDI ARABIA

## Abstract

Severe Fever with Thrombocytopenia Syndrome (SFTS) is an emerging infectious disease caused by a novel bunyavirus, SFTS virus (SFTSV), with fatal outcome developed in approximately 17% of the cases. Thrombocytopenia is a hallmark feature of SFTS, and associated with a higher risk of fatal outcome, however, the pathophysiological involvement of platelet in the clinical outcome of SFTS remained under-investigated. In the current study, by retrospectively analyzing 1538 confirmed SFTS patients, we observed that thrombocytopenia was associated with enhanced activation of the cytokine network and the vascular endothelium, also with a disturbed coagulation response. The platelet phenotypes were also extensively altered in the process of thrombocytopenia development of SFTS patients. More importantly, all these disturbed host responses were related to the severity of thrombocytopenia, thus were considered to play in a synergistic way to influence the disease outcome. Moreover, the clinical effect of platelet transfusion was assessed by comparing two groups of patients with or without receiving this therapy. As a result, we observed no therapy effect in altering frequencies of fatal outcome, clinical bleeding development, or dynamic change of platelet count during the hospitalization. It’s suggested that platelet supplementation alone acted a minor role in improving disease outcome, therefore new therapeutic intervention to regulate host response should be proposed. The current results revealed some evidence of interrelationship between platelet count and clinical outcome of SFTS disease from the perspective of activation of the cytokine network, the vascular endothelium, and the coagulation/fibrinolysis system. These evaluations might help to attain a better understanding of the pathogenesis and therapy choice in SFTS.

## Author summary

Thrombocytopenia in SFTSV is a multifactor-process involving a combination of platelet size or morphology alterations, fibrinolysis activation and coagulation abnormalities, increased inflammatory response and endothelial injury. Platelet supplementation alone shows minor role in improving disease, therefore new therapeutic intervention to regulate host response should be proposed.

## Introduction

Severe Fever with Thrombocytopenia Syndrome (SFTS) is an emerging infectious disease, with a wide distribution in 23 provinces in mainland China as well as in other Asian countries [1]. The causative pathogen is identified to be a novel bunyavirus, called SFTS virus (SFTSV) [2], which is transmitted through tick bite or by contact with blood or excretion of infected persons [3]. SFTSV genome consist of three single-stranded negative sense RNA segments, including large (L), medium(M) and small (S) segments. The S segment PCR amplification or SFTSV-specific antibodies was often used for SFTSV infection diagnosis or confirmation [4]. An extensively wide clinical spectrum was displayed from the SFTSV infected patients, mostly non-specific clinical manifestations, like fever, gastrointestinal symptoms, myalgia, and regional lymphadenopathy. The majority of cases experienced self-limiting clinical course, while fatal outcome developed in approximately 17% of the cases [5]. Except for supportive care, no specific therapies or drugs have proven to be effective clinically. Thrombocytopenia is a hallmark feature of SFTS, with its incidence ranging from 62% to 100% among hospitalized patients [6], which is higher than those reported from other viral hemorrhagic fever (VHF), such as Ebola disease or dengue hemorrhagic fever [7, 8]. Although low platelet counts were associated with and may contribute to mortality in SFTS patients [5], the mechanism underlying this relationship remained obscure. In more recent years, platelets are increasingly recognized for multiple biological functions in VHF pathogenesis, besides its role in participating in the process of hemostasis and thrombosis [9–11]. Although there are common pathogenesis to all VHF, the underlying mechanism differed in each specific disease. Whether thrombocytopenia acts as a significant central component of pathophysiology in SFTS disease remains to be determined. In the current study, we aimed to test the association between thrombocytopenia and systematic host response of SFTS from the perspective of cytokine network, the vascular endothelium, and the coagulation/fibrinolysis system. Moreover, the clinical effect of platelet transfusion was further assessed by comparing two groups of patients with or without receiving this therapy. These evaluations might help to attain a better understanding of the pathogenesis and therapy choice for SFTS.

## Materials and methods

### Patients recruitment and data collection

The study was designed to retrospectively analyze the data from confirmed SFTS patients who had been hospitalized in the 990 Hospital of Chinese People's Liberation Army Joint Logistic Support Force (PLA 990 hospital) in Xinyang, the most severely inflicted area in China [12]. All the confirmed SFTSV infection were recruited into the study, which had been defined by positive SFTSV RNA detection in blood samples that were collected on admission into hospital by TaqMan-based one-step real-time reverse transcriptase polymerase chain reaction (RT-PCR) assay [13]. A standard protocol was followed for the patients recruit, data collection and analysis. The patients’ information, including the clinical manifestations, the laboratory test and the therapy regimens, were collected using a standard medical recording form. The clinical symptoms and syndromes involving gastrointestinal system, respiratory system, neurological system, vascular and coagulation system, that were daily recorded were extracted from the medical record. Seven laboratory indicators that were tested throughout the hospitalization, including coagulation parameters, platelet count, activated partial thromboplastin time (APTT), thrombin time (TT), prothrombin time (PT), international normalized ratio (INR), fibrinogen (FIR) and D-dimer, were drawn from the medical database for further analysis.

The infection of HIV, tuberculosis or viral hepatitis had been tested in the designated hospital as routine laboratory test items, and these test results were also reviewed to exclude the coinfection. The other infectious disease that needed differential diagnosis, like dengue, hanta or malaria, had been ruled out by clinicians according to their exposure history, clinical manifestation and antibody detection.

### Patients exclusion and Data usage

From 2011 to 2016, altogether 1759 confirmed SFTS patients were hospitalized. Among them, 209 patients with delay of over 7 days between disease onset and hospital admission and 12 patients with missing data were excluded from the study. Therefore, the remaining 1538 patients were used for the final analysis on assessing the pathophysiological involvement of platelet in the clinical outcome of SFTS.

For the effect evaluation of platelet transfusion in improving thrombocytopenia, only the patients who had a nadir platelet count ≤30×10^9^/L were analyzed. Patients were excluded from analysis if they already had persistent or recurrent epistaxis, hematemesis, or melena on admission; or died within 48 h after hospitalization; or if they had no data on platelet counts available after therapy. Of the remaining patients, those who had been given platelet transfusion when their platelet count was reduced to ≤30×10^9^/L were defined as transfusion group, and the others who received no platelet transfusion throughout the hospitalization were classified into control group. For both groups, supportive therapy measures, including fluid therapy, parenteral nutrition therapy, fever and pain medications, et, were administered according to the same guideline [14]. The primary outcome was case fatality rate (CFR) that was obtained by prospectively following up the patients as previously described [5]; the secondary outcomes were the newly developed clinical bleeding and the change of platelet count that were evaluated on day 1, 2 and 3 following the platelet counts were reduced to 30×10^9^/L or lower.

### Sample storage and cytokine/adherence factor evaluation

For all the hospitalized patients, the serum samples had been collected, stored at -80°C and archived into the biological bank, which were used for the current cytokine assay. Briefly, the cytokine/chemokines were evaluated from the acute serum samples by the Bio-plex Pro Human 27-plex cytokine panel (Bio-Rad) following manufacturer’s instruction. Serum levels of vascular endothelial growth factor A (VEGF-A), P-selectin, E-selectin, platelet endothelial cell adhesion molecular (PECAM-1), CD40 ligand (CD40L), tissue plasminogen activator (tPA), plasminogen activator inhibitor 1 (PAI-1), serum amyloid antigen 1 (SAA-1), vascular cell adhesion molecular 1 (VCAM-1) and intercellular adhesion molecular 1 (ICAM-1) were determined by the ProcartaPlex multiplex immunoassays panels (Affymetrix, USA) according to the manufacturer instructions.

### Statistical analysis

For inter group comparison, the patients were classified into four groups according to their lowest platelet counts before treatment: platelet count ≤30×10^9^/L as the very low group (VLP), 30×10^9^/L~50×10^9^/L as the intermediate low group (ILP), 50×10^9^/L~100×10^9^/L as the low group (LP) and >100×10^9^/L as the normal group (NP). Within each group, frequency (percentage) was summarized for categorical variables, mean ± standard deviation (SD) was summarized for parametric quantitative variables, and median and interquartile range (IQR) was summarized for nonparametric quantitative variables. The intergroup comparison was made by applying independent *t*-test, *χ*^2^ test, Fisher exact test, analysis of variance, or nonparametric Mann-Whitney U test where appropriate. A multivariable Cox proportional hazard model was used to determine the association between differences in platelet counts on admission and mortality by day 30 when adjusted the variables of age, sex, and interval days from disease onset to admission. Cytokines and adherence factors were analyzed as log-transformed continuous variables to achieve their right-skewed distributions, and if not the nonparametric Mann-Whitney U test was used. Logistic regression model was performed to identify the factors that were associated with hemorrhagic manifestations or fatal outcome. Odds ratio (OR) and its 95% confidence interval (CI) were estimated. A 2-sided *P* value of <0.05 was considered to be statistically significant. Dynamic changes were analyzed over time with the generalized estimating equation (GEE). All analyses were performed using Stata 14.0 (Stata Corp LP, College Station, TX, USA).

The study protocol was approved by the Institutional Review Board of the Beijing Institute of Microbiology and Epidemiology and the PLA 990 hospital. Written informed consents were obtained from the patients or their guardians.

## Results

### Patient characteristics

Altogether 1538 patients (40.7% male, mean±SD for age: 60.8±12.4 years old) were used for the final analysis on assessing the pathophysiological involvement of platelet in the clinical outcome of SFTS. According to the platelet counts that were evaluated within 7 days after disease onset, 224 (14.6%) patients were classified into the VLP group, 531 (34.5%) in the ILP group, 681 (44.3%) in the LP group, and the remaining 102 (6.6%) in the NP group. Throughout disease progression, platelet count remained significantly different among four groups, with the most severe thrombocytopenia occurring between 6 to 8 days for all groups (S1 Fig).

### Platelet counts and disease outcome

**Fig 1:**
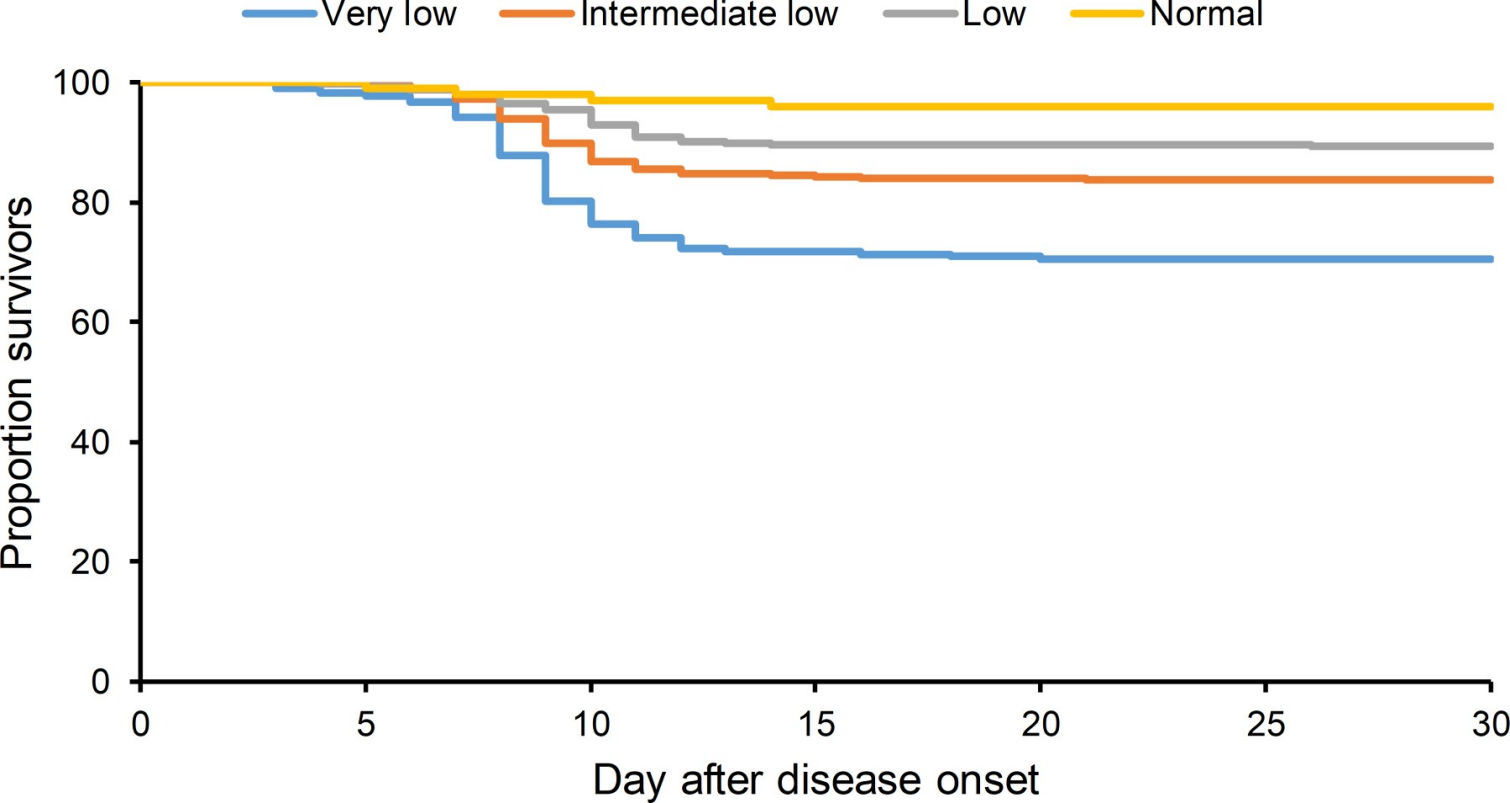
Kaplan-Meier survival curves of SFTS patients with different PLT counts. The patients were classified into four groups according to the lowest PLT counts within first 7 days after disease onset: Very Low indicates PLT count ≤30×10^9^/L, Intermediate low indicates PLT count 30×10^9^/L~50×10^9^/L, LP indicates PLT count 50×10^9^/L~100×10^9^/L, NP indicates PLT >100×10^9^/L.

The four groups of patients were compared for their baseline characteristics and underlying conditions to explore the factors that were related to the platelet decrease at the acute disease. It’s demonstrated that older age, male gender, preexisting hepatitis and chronic respiratory syndrome were significantly associated with more severe thrombocytopenia, which effect was also displayed by a dose dependent manner (Table 1). The four groups of patients differed regarding their main clinical manifestations and development of complication, in that headache, respiratory system syndrome (cough, sputum, dyspnea), gastrointestinal system syndromes (nausea), neurological system (blurred mind, convulsion) and bleeding (gingival bleeding) were recorded with higher frequency in the patients with VLP than the other groups. By performing multivariate analysis to adjust for the effect from demographic factors and preexisting conditions, the very low PLT counts were significantly associated with higher risk of severe complications than the patients from either of other three groups, manifested as increased incidence of bleeding and neurological syndromes, as well as resultant longer hospital duration (S1 Table). A significant dose effect between decreased platelet count and elevated CFR was also observed by performing multivariable Cox regression analysis (Fig 1). Patients of VLP and ILP groups showed an increased CFR compared with patients in the NP group (hazard ratios and 95% CI, 5.79 [2.10–15.96] and 3.03 [1.11–8.30]).

**Table 1:** Demographic and clinical characteristics of SFTS patients stratified by platelet counts.

Demographic Characteristics	PLT count level				P value
	Very low (n = 224)	Intermediate low (n = 531)	Low (n = 681)	Normal (n = 102)	
**Age, years, mean±SD**	64±9*^†^	62±11*^†^	59±13*	54±13	**<0.001**
**Sex, female, n (%)**	115 (51.3)	299 (56.3)	423 (62.1)	75 (73.5)	**<0.001**
**Delay from onset to admission, days, median (IQR)**	5 (4–6)	5 (4–6)	5 (4–6)	5 (4–7)	0.450
**Delay days>5, n (%)**	71 (31.5)	180 (33.9)	260 (38.2)	46 (45.1)	**0.050**
**Tick bite history, n (%)**	31 (13.8)	66 (12.4)	96 (14.1)	21 (20.6)	0.188
**Underlying diseases, n (%)**					
Cardiovascular & cerebrovascular diseases	44 (19.6)	100 (18.8)	134 (19.7)	20 (6.7)	0.985
Hypertension	16 (7.1)	47 (8.9)	71 (10.4)	13 (12.8)	0.307
Diabetes	14 (6.3)	33 (6.2)	42 (6.2)	2 (2.0)	0.380
Hepatitis	57 (25.5)	76 (14.3)	66 (9.7)	5 (4.9)	**<0.001**
Chronic respiratory disease	18 (8.0)	26 (4.9)	23 (3.4)	1 (1.0)	**0.008**
**Clinical characteristics**					
Fever	223 (99.6)	529 (99.6)	680 (99.9)	100 (98.0)	0.070
Lymphadenectasis	112 (50.0)	261 (49.2)	355 (52.1)	50 (49.0)	0.749
Myalgias	175 (78.1)	442 (83.2)	552 (81.1)	75 (73.5)	0.086
Weakness	218 (97.3)	520 (97.9)	668 (98.1)	96 (94.1)	0.092
Dizziness	56 (25.0)	110 (20.8)	130 (19.1)	21 (20.6)	0.307
Headache	45 (20.1)	64 (12.1)	82 (12.0)	16 (15.7)	**0.012**
Chills	28 (12.5)	43 (8.1)	81 (11.9)	15 (14.7)	0.070
Arthralgia	5 (2.2)	23 (4.3.)	27 (4.0)	2 (2.0)	0.400
Respiratory system	138 (61.6)	302 (56.9)	311 (45.7)	32 (31.4)	**<0.001**
Cough	130 (58.3)	296 (55.9)	295 (43.4)	32 (31.4)	**<0.001**
Expectoration	109 (48.9)	234 (44.2)	219 (32.2)	24 (23.5)	**<0.001**
Dyspnea	26 (11.7)	42 (7.9)	34 (5.0)	6 (5.9)	**0.006**
Gastrointestinal system	213 (95.1)	495 (93.2)	632 (92.8)	92 (90.2)	0.419
Abdominal pain	14 (6.3)	23 (4.3)	45 (6.6)	2 (2.0)	0.125
Nausea	161 (71.9)	369 (69.5)	481 (70.7)	55 (53.9)	**0.005**
Anorexia	178 (79.5)	387 (72.9)	480 (70.6)	78 (76.5)	0.060
Diarrhea	62 (27.7)	162 (30.5)	172 (25.3)	23 (22.6)	0.147
Vomit	85 (38.0)	184 (34.7)	242 (35.6)	31 (30.4)	0.597
Diarrhea & Vomit	122 (54.5)	264 (49.7)	331 (48.6)	42 (41.2)	0.154
Neurological system	20 (8.9)	39 (7.3)	29 (4.3)	1 (1.0)	**<0.001**
Coma	2 (0.9)	8 (1.5)	3 (0.4)	0 (0)	0.173
Dysphoria	5 (2.2)	11 (2.1)	8 (1.2)	0 (0)	0.280
Lethargy	0 (0)	0 (0)	1 (0.2)	0 (0)	0.739
Blurred mind	12 (5.4)	27 (5.1)	21 (3.1)	0 (0)	**0.035**
Convulsion	9 (4.0)	20 (3.8)	8 (1.2)	1 (1.0)	**0.009**
Bleeding	19 (8.5)	27 (5.1)	26 (3.8)	4 (3.9)	**0.045**
Melena	3 (1.3)	7 (1.3)	5 (0.7)	0 (0)	0.500
Gingival Bleeding	7 (3.1)	6 (1.1)	2 (0.3)	0 (0)	**0.002**
Haemoptysis	1 (1.0)	0 (0)	2 (0.3)	1 (1.0)	0.289
Haematemesis	1 (0.5)	2 (0.4)	1 (0.2)	0 (0)	0.758
Epistaxis	1 (0.5)	0 (0)	0 (0)	0 (0)	0.212
Macroscopic haematuria	5 (2.2)	9 (1.7)	6 (0.9)	3 (2.9)	0.245
Ophthalmorrhagia/conjunctival congestion	0 (0)	0 (0)	3 (0.4)	0 (0)	0.505
Ecchymosis/petechial	5 (2.2)	7 (1.3)	7 (1.0)	0 (0)	0.387

The patients were classified into four groups according to the lowest platelet count within first 7 days after disease onset: platelet count ≤30×10^9^/L as the very low group (VLP), 30×10^9^/L~50×10^9^/L as the intermediate low group (ILP), 50×10^9^/L~100×10^9^/L as the low group (LP) and >100×10^9^/L as the normal group (NP).

†P<0.05 compared to the low group

‡P<0.05 compared to the intermediate low group.

### Platelet counts and platelet size parameters, endothelial activation, and coagulation perturbation

**Fig 2:**
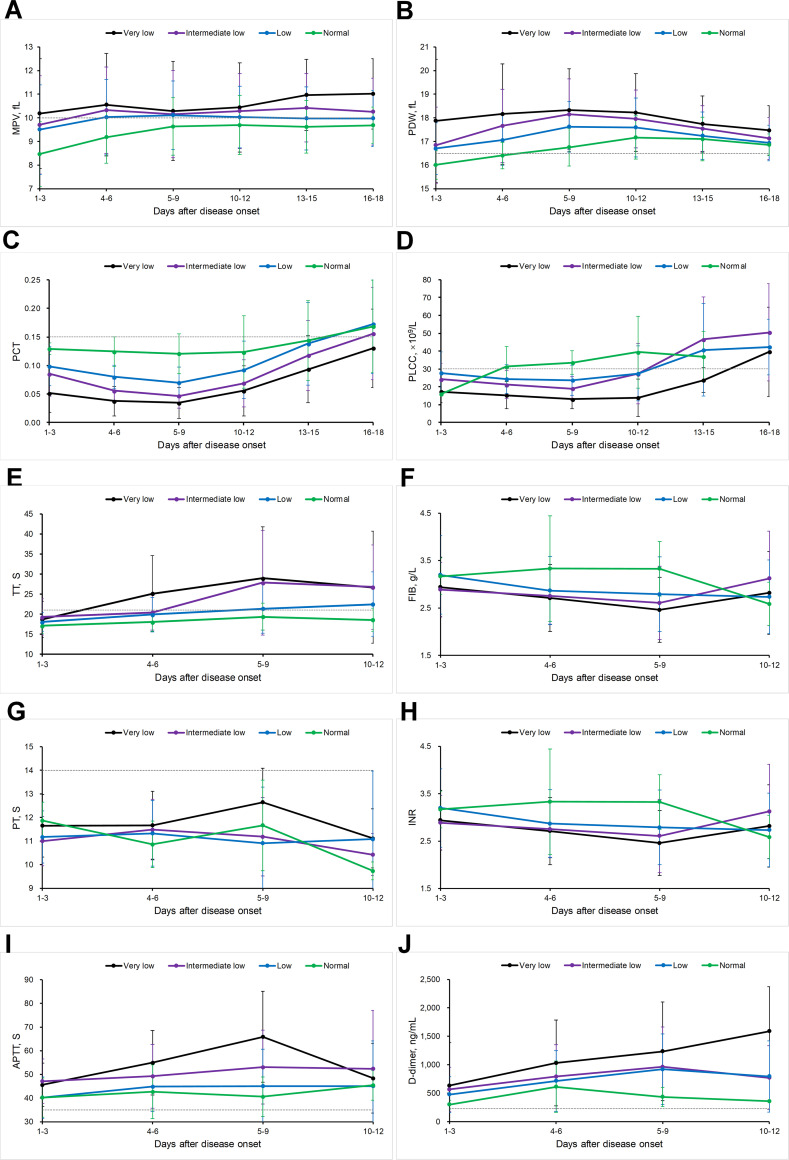
The profiles of platelet related laboratory parameters in the four groups with different PLT counts. A, MPV, mean platelet volume; B, PDW, platelet distribution width; C, PCT, thrombocytocrit; D, PLCC, platelet large cell counts. E, TT, thromboplastin time; F, FIB, fibrinogen; G, PT, prothrombin time; H, INR, international normalized ratio; I, APTT, activated partial thromboplastin time; J, D-dimer. The mean and standard deviation were shown.

Platelet volume indices are useful in assessing the etiology of thrombocytopenia [15]. Altogether four platelet size parameters were evaluated throughout the hospitalization duration, which significantly differed among four groups. Mean platelet volume (MPV) and platelet distribution width (PDW) were increased, whereas the plateletcrit (PCT) and platelet large cell count (PLCC) were decreased, all corresponded with the declination of platelet count (Fig 2A–2D).

**Fig 3:**
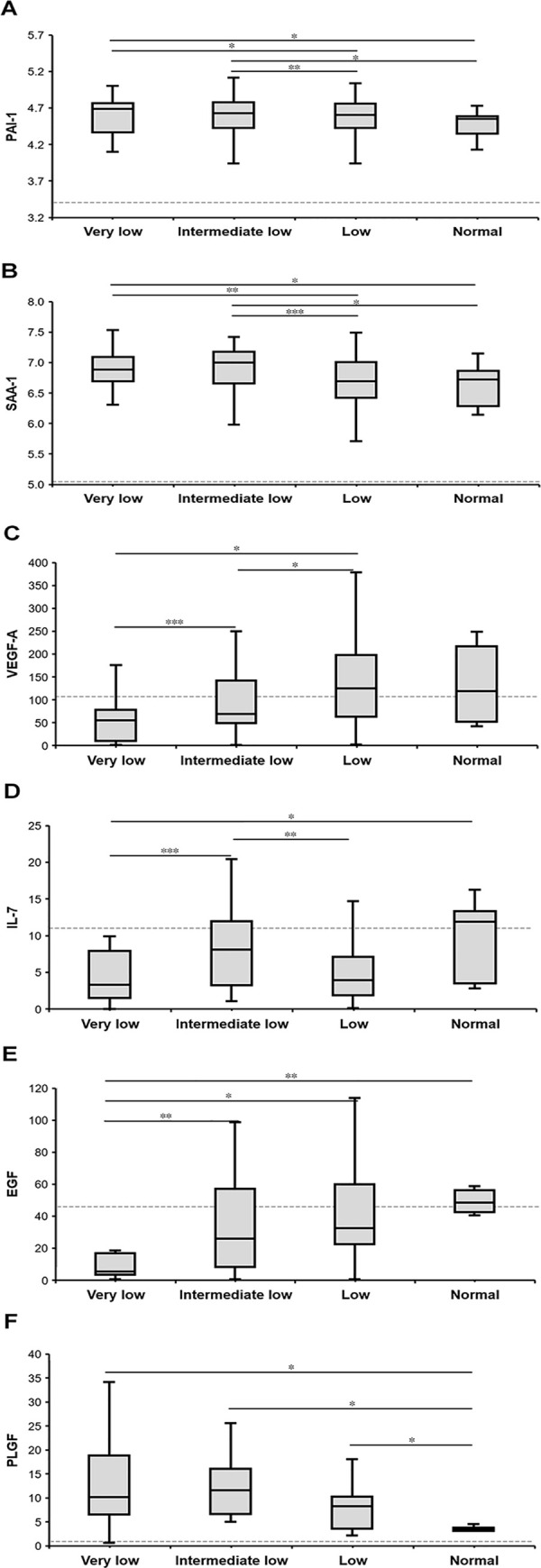
Cytokine levels in SFTS patients in the four groups with different PLT counts at the acute phase. A, PAI-1, plasminogen activator inhibitor 1; B SAA-1, serum amyloid antigen 1; C, VEGF-A, vascular endothelial growth factor A; D, IL-7, interleukin 7; E, EGF, epidermal growth factor; F, PLGF, placental growth factor. *P<0.05 using nonparametric test; **P<0.01 using nonparametric test; ***P<0.001 using nonparametric test. Data are expressed as box plots with the median, lower quartile, upper quartile, minimum and maximum.

Five coagulation factors (TT, FIB, PT, INR, and APTT) were evaluated throughout the hospitalization (Fig 2E–2I). Except for PT, all the other four indicators showed inter group difference that was consistent with the platelet count. More deviation from the normal value was observed in the ILP and VLP group than the other two groups, especially at 5–9 days after disease onset. This is exactly the time period when the clinical deterioration occurred in fatal cases, thus indicating a significant association between severity of thrombocytopenia and risk of extrinsic coagulation disturbance and subsequent disseminated intravascular coagulation development. The markers of fibrinolysis activation, PAI-1, D-dimer and SAA-1 [16], were increased at acute SFTSV infection, and more importantly, were elevated to an even higher level in the VLP and ILP groups, in comparison with either LP or NP group (Fig 3A and 3B). D-dimer exclusively showed a clear correlation with platelet levels (Fig 2J).

Our previous research has displayed an obvious activation of the vascular endothelium in SFTS patients as a general group [17]. Here when the SFTS patients were grouped according to PLT counts, four adherence molecules (VEGF-A, IL-7, EGF and PLGF) demonstrated inter group difference, especially VEGF-A and EGF were decreased in SFTS patients, showing dose effect on the platelet counts, with their concentrations inversely correlated with the platelet counts (Fig 3C–3F).

### Effect of platelet transfusion on the alteration of bleeding

As the patients with platelet counts decreased below 30×10^9^/L had highest risk of death, we further assessed the effect of prophylactic platelet transfusion in reversing the process of thrombocytopenia and altering the disease outcome in this group. Among 1759 confirmed SFTS patients, 35 patients without the continuous data of platelet count, 65 patients with hospital discharge within 2 days and 46 patients with persist bleeding on admission were excluded. Therefore, the remaining 1613 patients were used for the final analysis on assessing clinical effect of platelet transfusion (S2 Fig). Altogether 250 patients who were given platelet transfusion with a median of 2 (range 1–3) units of platelets and 72 patients who received no transfusion that met the inclusion criteria were used for the effect evaluation.

**Fig 4:**
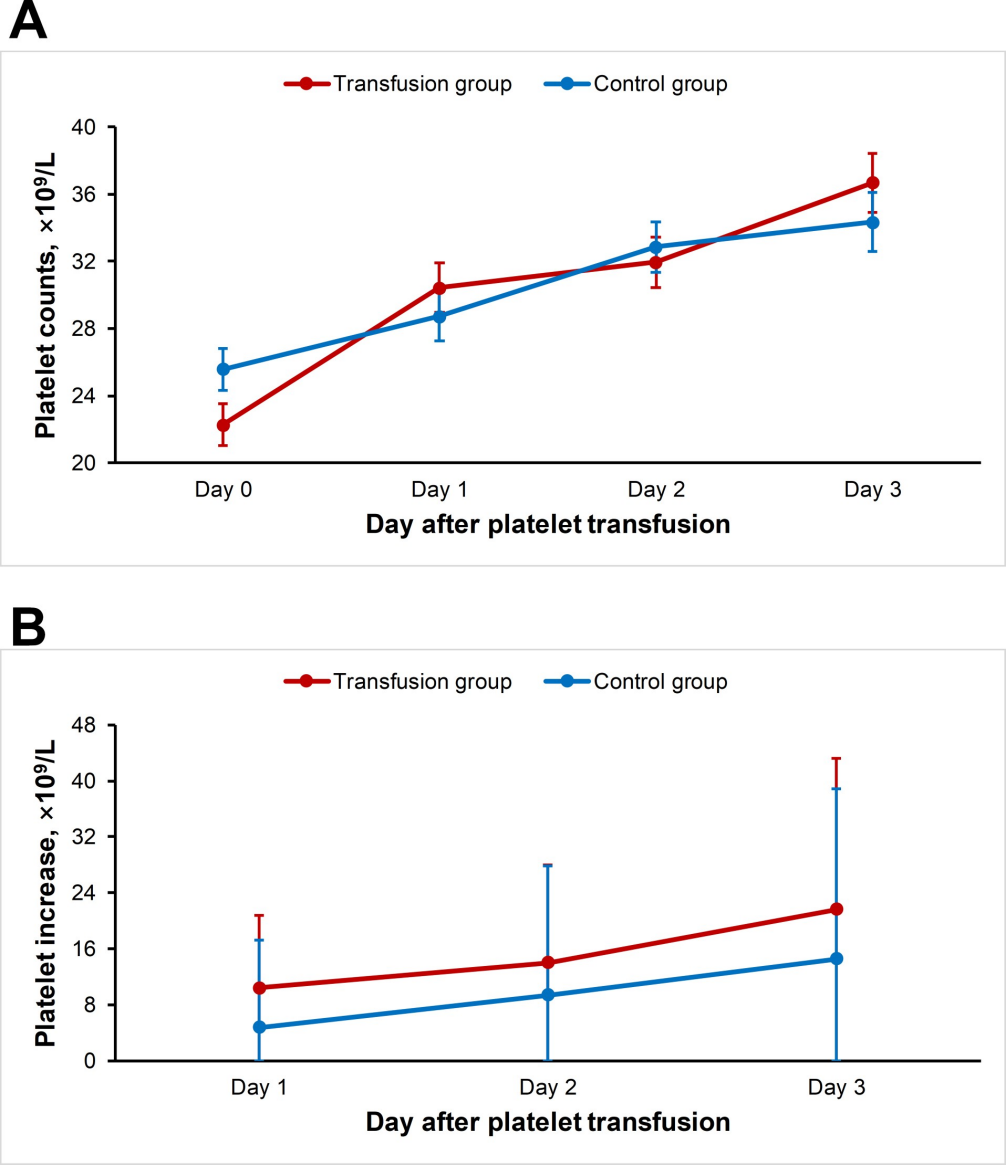
The change of platelet counts after platelet transfusion in the SFTS patients. A, the platelet counts evaluated at Day 1, 2 and 3 after the platelet transfusion; B, the change value of platelet counts evaluated at Day 1, 2 and 3 after the platelet transfusion. The mean and standard deviation were shown.

The baseline demographic data and clinical features on admission into the hospital were comparable between groups (S2 Table). When hospitalization duration and the development of fatal outcome were used as outcome, no significant difference was attained between two groups, after adjusting the effect from age, sex, delay, diabetes, hypertension and hepatitis (P = 0.266 and P = 0.287). We also used other outcomes to measure the effect of platelet transfusion, i.e., the occurrence of clinical bleeding up to hospital discharge, and the change of platelet count at day 1, day 2, and day 3 after transfusion (S3 Table). No statistical difference of the occurrence of clinical bleeding between patients who were given prophylactic transfusion or patients without prophylactic transfusion (29.6% vs 22.2%, P = 0.225) By applying the mixed effect model, the median elevation of platelet counts after 3-day platelet transfusion was comparable with the natural increment value of the platelet count that were calculated on 3 days after the platelet were reduced below 30×10^9^/L in patients without receiving transfusion (β = -3.61; 95% CI -8.38, 1.16; P = 0.138) (Fig 4). Although the one-day transfusion slightly increased the platelet counts, the increment value attained no significance after adjusting the above variables (β = 5.50; 95% CI 0.35–11.35; P = 0.065). When the data were further separated into four clinical courses, no significant differences of platelet counts were found between the two groups during any of the clinical courses (S3 Fig).

## Discussion

VHFs are a diverse group of zoonotic and human illnesses in which fever and bleeding are hallmark clinical features [18]. Although the viral pathogens varied widely, they share some common features of the clinical manifestation and disease progression, with thrombocytopenia as one of the features [19]. As it has been demonstrated in SFTS, a newly recognized VHF disease, severe thrombocytopenia occurred, which in combination with depressed immune responses, composed two prominent features that underlie the SFTS related death [20]. Although previous evidence indicating that platelets can influence disease outcome is abundant, clinical studies addressing key host responses in thrombocytopenia are limited. In the current research, by retrospectively analyzing biomarkers indicative of activation or dysregulation of host response pathways relevant for SFTS, we have provided evidence that thrombocytopenia is associated with enhanced activation of the cytokine network and endothelia cells, and with a disturbed coagulation response. The platelet function and phenotypes were also extensively comprised in the process of thrombocytopenia. More importantly, all these disturbed host responses were related to the severity of thrombocytopenia, thus were considered to play in a synergistic way to influence the disease outcome. Taken this into account, it’s reasonable to observe that mere supplementation of platelet played only minor role in improving the clinical phenotype and outcome, as thrombocytopenia in SFTS is a multifactor-process involving a combination of platelet function alterations, fibrinolysis activation and coagulation abnormalities, increased inflammatory response, endothelial injury and platelet aggregation. We believe this is not against common sense, some studies had even proved the platelet transfusion might be detrimental more than beneficial in infectious disease [21].

As the most directly involved system in VHFs, coagulation and fibrinolysis were also disturbed with acute SFTSV infection, akin to the situation in Ebola and dengue [19]. Plasma PT and APTT are indexes of extrinsic and intrinsic coagulation system during the coagulation cascade, respectively [22]. In the current research, prolongation of both indicators occurred in all thrombocytopenia groups, with more profound abnormalities in the severe thrombocytopenia group. All these findings suggested a perturbed extrinsic pathway in SFTS patients and an even stronger activation of the intrinsic pathway in the severe cases [16]. Increased concentrations of D-dimers and tPA indicated the activation of fibrinolysis, which is believed to be related to disseminated intravascular coagulation [23], a feature commonly seen in the severest stage of SFTSV infection.

As it has been displayed in our previous research, the vascular endothelial could act as a potential target by SFTSV infection [24], which is also the prime site initiating the systematic inflammation response in other life-threatening infections [25]. Besides, the vascular endothelial system could interact with platelet through several pathways [26]. Here by evaluating ten inflammatory cytokines indicative of endothelial function, we found four cytokines had their concentrations significantly associated with the extent of thrombocytopenia. VEGF-A is a potent mediator for angiogenesis and vascular remodeling, which would undergo severe reduction during inflammatory injury [27], while PLGF could abolish the regulatory effect of VEGF-A isoforms [28]. IL-7R was expressed in vascular endothelial, and both recombinant IL-7 and EGF has shown therapeutic effect on endothelial dysfunction [29, 30]. Based on these results, we suggest the homeostasis of vascular endothelial cells have interaction with platelet, participating in the process of the platelet destruction, finally contributing to the adverse disease outcome.

Prior to the complete understanding of pathogenesis mechanism of SFTSV infection, the therapy regimens were limited to antiviral therapy to reduce viral load, corticosteroid therapy to relieve inflammatory response, and platelet transfusion in case of clinical bleeding. However, the current findings supported no efficacy of platelet transfusion in improving the clinical phenotype or outcome, based on well balanced groups of patients receiving platelet transfusion or not. Based on the multiple pathways that were involved in the thrombocytopenia, we propose that other therapy choice, including strategies in reducing platelet adhesion and aggregation, as well as attenuating the inflammatory component of activated endothelial, should be applied. On the other hand, given the previous evidence from other VHF, such as in dengue, prophylactic platelet transfusion was not superior to supportive care in preventing bleeding, and might be even associated with adverse events [21], the usage of platelet transfusion for the SFTSV infection therapy should be cautiously applied, even for those patients with low platelet counts.

The study was subject to major limitation that although dose-effect relationship were observed, the causal relationship between platelet counts and the host immune response cannot be inferred only based on the association study. Whether thrombocytopenia is one of the causations of systematic host response, or only a phenotype accompanying SFTSV infection related human response requires further clinical and experimental data to verify.

In conclusion, it’s suggested that thrombocytopenia in SFTS is a multifactor-process that included but not limited to coagulation abnormalities, increased inflammatory response and endothelial activation. The platelet supplementation had no role in improving disease, and new therapeutic intervention that target human host response should be proposed. The current study could help to understand the mechanism underlying SFTS related thrombocytopenia, and might also provide a common mechanism underlying other VHF viruses’ infection.

## Supporting information

S1 Table The outcome of the patients with the four groups. (DOCX)

S2 Table The characteristics of SFTS patients on admission in the two groups of prophylactic platelet transfusion plus supportive care and supportive care alone. (DOCX)

S3 Table. The comparison of clinical outcomes between prophylactic platelet transfusion plus supportive care and supportive care alone (DOCX)

S1 Fig The dynamic profile of platelet counts over time in the four groups classified by the minimum platelet count within first 7 days after disease onset. The mean and standard deviation were shown. Very Low indicates PLT count ≤30×10^9^/L, Intermediate low indicates PLT count 30×10^9^/L~50×10^9^/L, LP indicates PLT count 50×10^9^/L~100×10^9^/L, NP indicates PLT >100×10^9^/L.
(TIF)

S2 Fig The flow diagram of recruiting patients for the efficiency evaluation of platelet transfusion. (TIF)

S3 Fig The dynamic profile of platelet counts along the days after the platelet transfusion that was administered at various stage. A, during the disease course; B, at Day 1–4 days after disease onset; C, at Day 5–7 days after disease onset; D, at Day 8–10 days after disease onset; E, at Day>10 days after disease onset. The red line presents for the transfusion group; the blue line presents for control group.
(TIF)
