# Supplementary material for: Correlation between thrombocytopenia and host response in severe fever with thrombocytopenia syndrome
Source: PLoS Negl Trop Dis. 2020 Oct 29;14(10):e0008801. doi: 10.1371/journal.pntd.0008801 (PMC7595704; doi:10.1371/journal.pntd.0008801)
Supplement: S1 Table — (DOCX) [file pntd.0008801.s001.docx]

S1 Table. The outcome of the patients with the four groups

| Characteristics | Very low  (n=276) | Intermediate low (n=636) | Low (n=827) | Normal (n=112) | P value | |
| --- | --- | --- | --- | --- | --- | --- |
| **Hospital duration, days, median (IQR)** | 10 (5-12) | 10 (8-12) | 9 (7-10) | 7 (5-10) | <0.001 | |
| **Neurological system** | 76 (35.7) | 103 (19.4) | 100 (15.1) | 5 (5.0) | <0.001 |  |
| Coma | 29 (13.1) | 33 (6.3) | 28 (4.1) | 0 (0) | <0.001 |  |
| Dysphoria | 48 (21.9) | 58 (11.2) | 53 (7.9) | 2 (2.0) | <0.001 |  |
| Lethargy | 22 (9.8) | 24 (4.5) | 21 (3.1) | 0 (0) | <0.001 |  |
| Blurred mind | 51 (24.1) | 64 (12.7) | 57 (8.6) | 5 (4.9) | <0.001 |  |
| Convulsion | 40 (18.6) | 49 (9.6) | 56 (8.3) | 0 (0) | <0.001 |  |
| **Bleeding** | 70 (33.3) | 88 (17.3) | 66 (10.0) | 2 (2.0) | <0.001 |  |
| Melena | 26 (11.8) | 35 (6.7) | 21 (3.1) | 0 (0) | <0.001 |  |
| Gingival Bleeding | 41 (18.9) | 42 (8.0) | 39 (5.7) | 1 (1.0) | <0.001 |  |
| Haemoptysis | 10 (4.5) | 14 (2.6) | 11 (1.6) | 1 (1.0) | 0.087 |  |
| Haematemesis | 8 (3.6) | 10 (1.9) | 6 (0.9) | 0 (0) | 0.019 |  |
| Epistaxis | 5 (2.2) | 2 (0.4) | 0 (0) | 0 (0) | 0.001 |  |
| Macroscopic haematuria | 0 (0) | 1 (0.2) | 1 (0.2) | 0 (0) | 0.902 |  |
| Ophthalmorrhagia/conjunctival congestion | 2 (0.9) | 1 (0.2) | 1 (0.2) | 0 (0) | 0.310 |  |
| Ecchymosis/petechial | 0 (0) | 0 (0) | 0 (0) | 0 (0) | 1.000 |  |
| **Fatal outcome** | 66 (29.5) | 86 (16.2) | 72 (10.6) | 4 (3.9) | <0.001 |  |
