# Supplementary material for: Correlation between thrombocytopenia and host response in severe fever with thrombocytopenia syndrome
Source: PLoS Negl Trop Dis. 2020 Oct 29;14(10):e0008801. doi: 10.1371/journal.pntd.0008801 (PMC7595704; doi:10.1371/journal.pntd.0008801)
Supplement: S2 Table — (DOCX) [file pntd.0008801.s002.docx]

**Table 1. The characteristics of SFTS patients on admission in the two groups of prophylactic platelet transfusion plus supportive care and supportive care alone**

| Characteristics | Transfusion group (n=250) | Control group (n=72) | *P* |
| --- | --- | --- | --- |
| Demographic characteristics |  |  |  |
| Male gender/ No. (%) | 114 (45.6) | 40 (55.7) | 0.264 |
| Age, year, mean±SD | 64±9 | 64±10 | 0.501 |
| Days delay, median (IQR) | 5 (4-7) | 5 (4-7) | 0.776 |
| Presence of underlying conditions, No. (%) of Patients | |  |  |
| Diabetes | 25 (10.0) | 5 (6.9) | 0.432 |
| Hypertension | 13 (5.2) | 6 (8.3) | 0.320 |
| Hepatitis | 56 (22.4) | 9 (12.5) | 0.065 |
| Clinical manifestations |  |  |  |
| Fever >38 ºC | 250 (100) | 71 (98.6) | 0.062 |
| Nervous system |  |  |  |
| Convulsion | 3 (1.2) | 0 (0) | 1.000 |
| Blurred mind | 6 (2.4) | 4 (5.6) | 0.239 |
| Lethargy | 0 (0) | 1 (1.4) | 0.224 |
| Dysphoric | 3 (1.2) | 2 (2.8) | 0.311 |
| Coma | 1 (0.4) | 0 (0) | 1.000 |
| Gastrointestinal syndromes |  |  |  |
| Vomit | 113 (45.2） | 23 (31.9) | 0.045 |
| Nausea | 197 (78.8) | 49 (68.1) | 0.059 |
| Diarrhea | 71 (28.4) | 29 (40.3) | 0.055 |
| Anorexia | 207 (82.8) | 62 (86.1) | 0.504 |
| Abdominal pain | 10 (5.7) | 8 (14.3) | 0.037 |
| Feeble | 243 (97.2) | 71 (98.6) | 0.498 |
| Myalgias | 207 (82.8) | 55 (76.4) | 0.218 |
| Lymphadenectasis | 127 (50.8) | 46 (63.9) | 0.050 |
| Arthralgias | 8 (3.2) | 3 (4.2) | 0.691 |
| Respiratory syndromes |  |  |  |
| Cough | 155 (62.3) | 42 (58.3) | 0.548 |
| Sputum production | 133 (53.4) | 34 (47.2) | 0.354 |
| Dyspnea | 33 (13.4) | 8 (11.1) | 0.616 |
| Dizzy | 61 (24.4) | 19 (26.4) | 0.731 |
| Headache | 48 (19.2) | 12 (16.7) | 0.627 |
| Chill | 24 (9.6) | 15 (20.8) | 0.010 |
| Laboratory features on admission |  |  |  |
| WBC<4.0×10^9^/L | 208 (83.2) | 57 (19.2) | 0.429 |
| PLT<100×10^9^/L | 243 (97.2) | 71 (98.6) | 0.498 |
| Neutrophils>70% | 126 (50.) | 40 (55.6) | 0.441 |
| Lymphocytes<20% | 104 (41.6) | 30 (41.7) | 0.992 |
| HGB<110g/L | 56 (22.4) | 12 (16.7) | 0.294 |
| AST>40U/L | 231 (92.4) | 68 (94.4) | 0.553 |
| ALT>40U/L | 171 (68.4) | 55 (76.4) | 0.192 |
| ALB<35g/L | 84 (34.3) | 19 (29.2) | 0.442 |
| ALP>150U/L | 17 (6.8) | 8 (11.1) | 0.228 |
| GGT>50U/L | 65 (26.0) | 21 (29.2) | 0.593 |
| LDH>245U/L | 225 (90.0) | 66 (91.7) | 0.637 |
| CK>200U/L | 170 (68.3) | 60 (83.3) | 0.013 |
| BUN>7.14mmol/L | 112 (46.1) | 29 (48.3) | 0.755 |
| TBIL>17.1mmol/L | 30 (12.0) | 8 (11.1) | 0.837 |
| CREA>97μmol/L | 66 (26.4) | 16 (22.4) | 0.473 |
| AMY>115U/L | 63 (42.0) | 16 (39.0) | 0.732 |

Note: SD, standard deviation; IQR, interquartile range.
