# Supplementary material for: Correlation between thrombocytopenia and host response in severe fever with thrombocytopenia syndrome
Source: PLoS Negl Trop Dis. 2020 Oct 29;14(10):e0008801. doi: 10.1371/journal.pntd.0008801 (PMC7595704; doi:10.1371/journal.pntd.0008801)
Supplement: S3 Table. The comparison of clinical outcomes between prophylactic platelet transfusion plus supportive care and supportive care alone — (DOCX) [file pntd.0008801.s003.docx]

**S3 Table. The comparison of clinical outcomes between** **prophylactic platelet transfusion plus supportive care and supportive care alone**

| Outcome | Platelet transfusion | |  | Crude | | |  | Adjusted* | | |
| --- | --- | --- | --- | --- | --- | --- | --- | --- | --- | --- |
|  | Yes | No |  | OR | 95%CI | P value |  | OR | 95%CI | P value |
| **Hemorrhage** |  |  |  |  |  |  |  |  |  |  |
| Yes | 74 (29.6) | 16 (22.2) |  | 1.472 | 0.793-2.731 | 0.221 |  | 1.475 | 0.787-2.765 | 0.225 |
| No | 176 (70.4) | 56 (77.8) |  |  |  |  |  |  |  |  |
| **Severe hemorrhage** |  |  |  |  |  |  |  |  |  |  |
| Yes | 32 (42.1) | 7 (43.8) |  | 0.935 | 0.315-2.775 | 0.904 |  | 1.262 | 0.383-4.165 | 0.702 |
| No | 44 (57.9) | 9 (56.2) |  |  |  |  |  |  |  |  |
| **Fatal** |  |  |  |  |  |  |  |  |  |  |
| Yes | 57 (22.8) | 12 (16.7) |  | 1.477 | 0.743-2.934 | 0.266 |  | 1.483 | 0.718-3.062 | 0.287 |
| No | 193 (77.2) | 60 (83.3) |  |  |  |  |  |  |  |  |
| **Hospital duration, median (IQR)** | 11 (7-13) | 10 (8-12) |  | 1.680 | 0.565-4.995 | 0.351 |  | 1.849 | 0.625-5.469 | 0.266 |

*The adjusted variables were age, sex, delay, diabetes, hypertension and hepatitis.
