# Supplementary figures and images for: Correlation between thrombocytopenia and host response in severe fever with thrombocytopenia syndrome
Source: PLoS Negl Trop Dis. 2020 Oct 29;14(10):e0008801. doi: 10.1371/journal.pntd.0008801 (PMC7595704; doi:10.1371/journal.pntd.0008801)

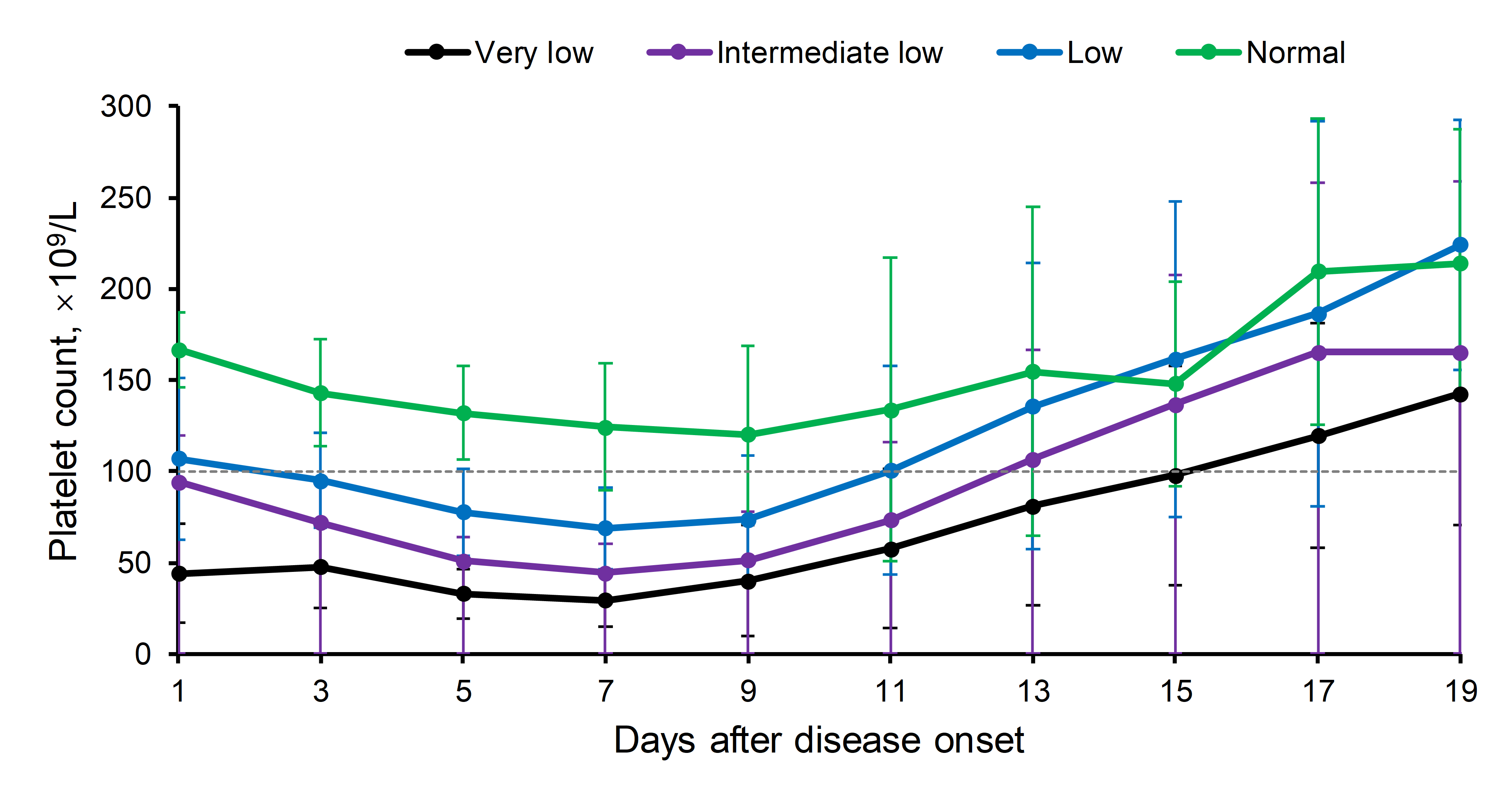

Supplement: S1 Fig — The mean and standard deviation were shown. Very Low indicates PLT count ≤30×109/L, Intermediate low indicates PLT count 30×109/L~50×109/L, LP indicates PLT count 50×109/L~100×109/L, NP indicates PLT >100×109/L. (TIF) [file pntd.0008801.s004.tif]

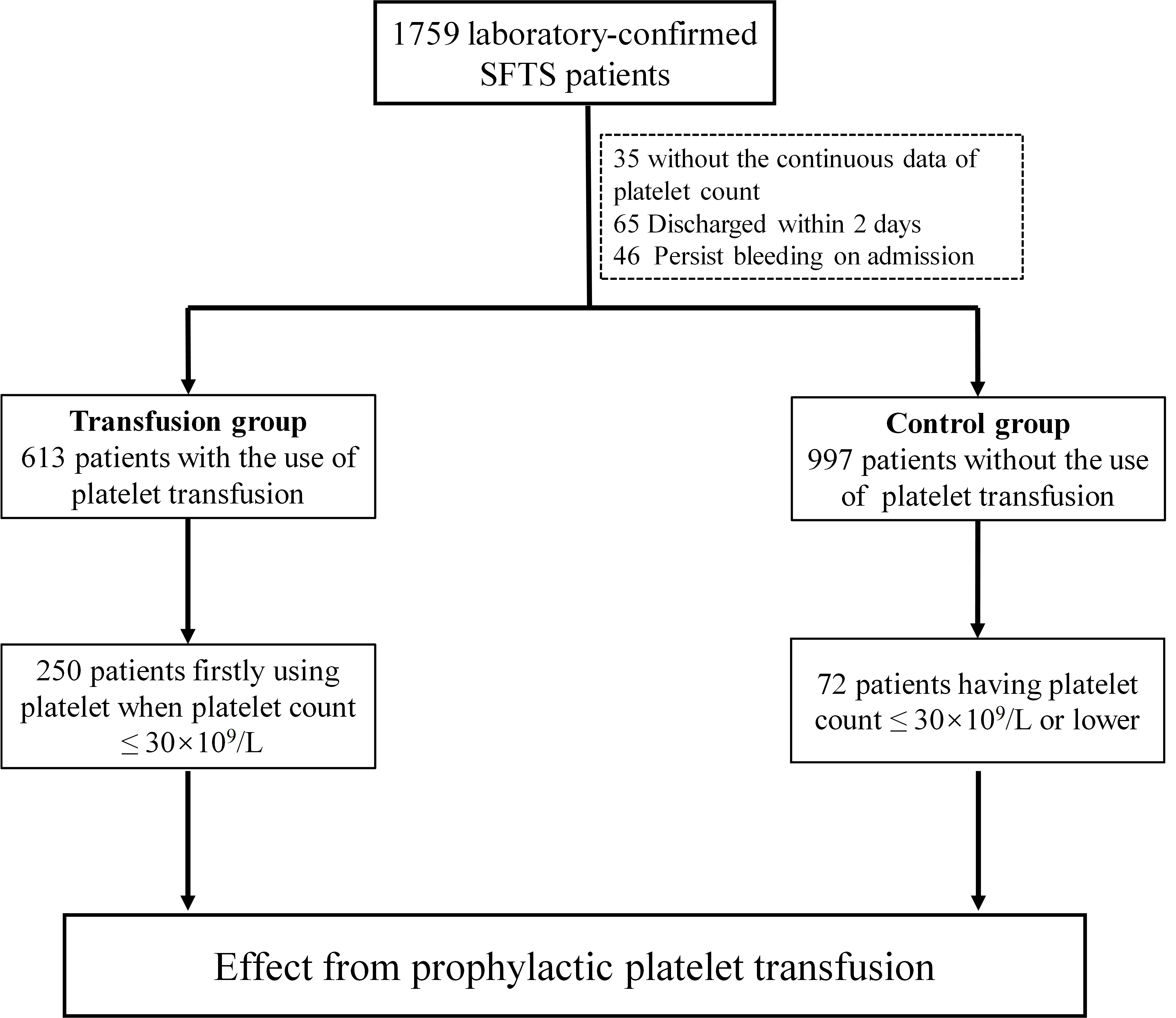

Supplement: S2 Fig — (TIF) [file pntd.0008801.s005.tif]

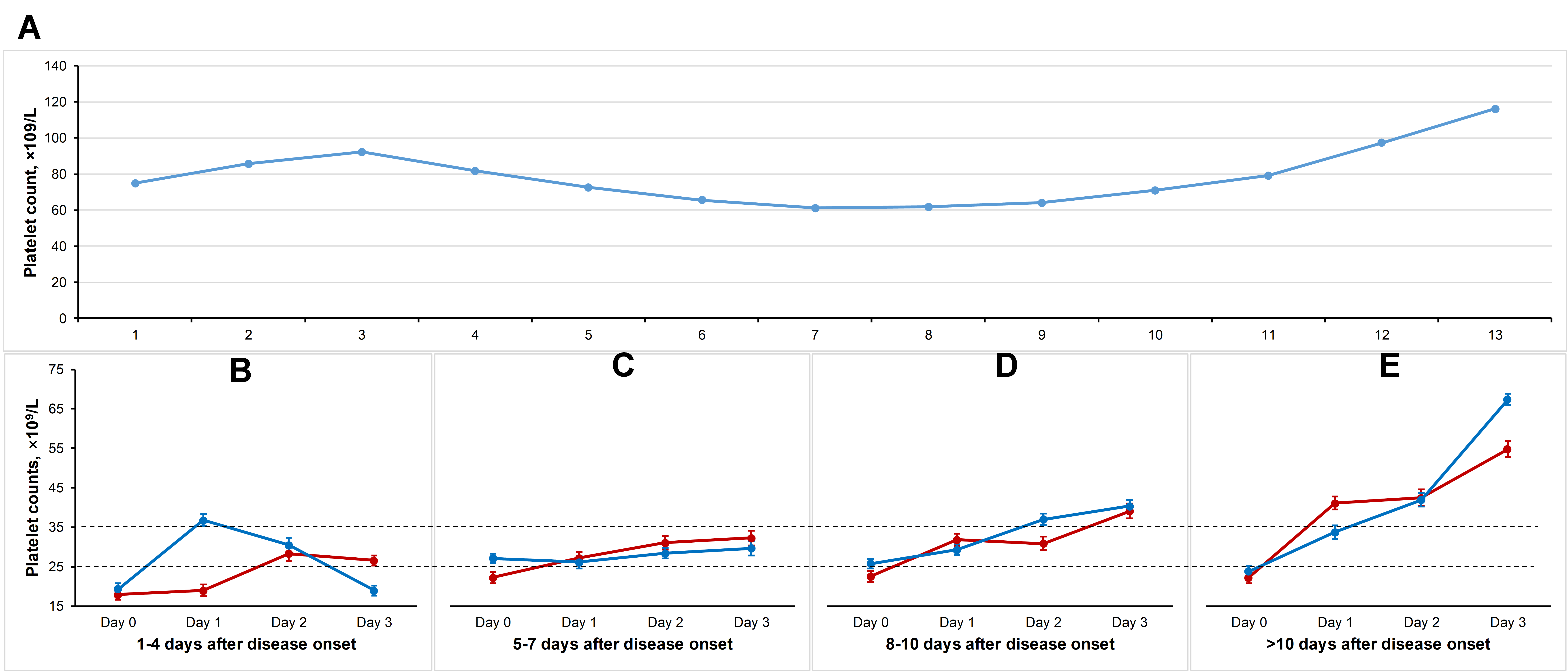

Supplement: S3 Fig — A, during the disease course; B, at Day 1–4 days after disease onset; C, at Day 5–7 days after disease onset; D, at Day 8–10 days after disease onset; E, at Day>10 days after disease onset. The red line presents for the transfusion group; the blue line presents for control group. (TIF) [file pntd.0008801.s006.tif]
